# Defect-Tolerant Memristor Crossbar Circuits for Local Learning Neural Networks

**DOI:** 10.3390/nano15030213

**Published:** 2025-01-28

**Authors:** Seokjin Oh, Rina Yoon, Kyeong-Sik Min

**Affiliations:** School of Electrical Engineering, Kookmin University, Seoul 02707, Republic of Korea; ghj163@kookmin.ac.kr (S.O.); flsk0419@kookmin.ac.kr (R.Y.)

**Keywords:** defect-tolerant memristor circuits, local learning, neural networks, equilibrium propagation

## Abstract

Local learning algorithms, such as Equilibrium Propagation (EP), have emerged as alternatives to global learning methods like backpropagation for training neural networks. EP offers the potential for more energy-efficient hardware implementation by utilizing only local neuron information for weight updates. However, the practical implementation of EP using memristor-based circuits has significant challenges due to the immature fabrication processes of memristors, resulting in defects and variability issues. Previous implementations of EP with memristor crossbars use two separate circuits for the free and nudge phases. This approach can suffer differences in defects and variability between the two circuits, potentially leading to significant performance degradation. To overcome these limitations, in this paper, we propose a novel time-multiplexing technique that combines the free and nudge phases into a single memristor circuit. Our proposed scheme integrates the dynamic equations of the free and nudge phases into one circuit, allowing defects and variability compensation during the training. Simulations using the MNIST dataset demonstrate that our approach maintains a 92% recognition rate even with a 10% defect rate in memristors, compared to 33% for the previous scheme. Furthermore, the proposed circuit reduces area overhead for both the memristor circuit solving EP’s algorithm and the weight-update control circuit.

## 1. Introduction

Backpropagation-based learning has demonstrated remarkable neural network performance in various applications such as vision, speech recognition, and so on [1,2,3,4,5]. The backpropagation is a kind of global learning algorithm. It means that the entire network’s information should be considered in calculating every single weight. This is one reason why the backpropagation algorithm needs a massive amount of computation, consuming a lot of computing energy to calculate the weight updates [6,7,8].

As an alternative, a local learning algorithm can be used to train neural networks [9,10]. Here, every single synapse can be updated using only its local neuron information. If one synapse is placed between two neurons, the two neurons can be local neurons for the synaptic weight. By using only local neuron information to update the weight between them, the local learning algorithm can reduce the amount of computation dramatically compared to global learning. Spike time-dependent plasticity (STDP) can be considered one example of local learning algorithms, where the weights are potentiated or depressed according to a simple Hebbian learning rule using only local neurons’ timing information [11,12,13,14]. Though the STDP’s hardware implementation can be much simpler than the hardware of backpropagation, the neural network’s performance trained by the STDP is still too low to be used in real AI applications [15,16].

Recently, Equilibrium Propagation (EP) has been proposed to train neural networks based on the local learning concept [10,12,17]. The EP algorithm works in two phases, which are the free phase and the nudge phase, respectively [18,19]. In the EP algorithm, a new concept of energy is defined using the traditional Hopfield network’s energy equation [20,21]. The network’s state variables in the EP algorithm are assumed to change dynamically with increasing time according to Equation (1) [12]:

(1) $$\frac{ds}{dt}=-\frac{\partial E}{\partial s}$$

Here, the variables s and E in Equation (1) mean the network’s state variable and energy, respectively. The state variable can reach a stable point when the network’s energy is minimized. Mathematically, the stable point of the state variable s can be calculated by solving the state variable’s dynamic Equation (1). However, if we try to solve the EP’s dynamic equation using digital CMOS hardware, the computing hardware that should be very complex may consume a large amount of computing energy [22]. This makes local learning useless in terms of energy efficiency and hardware complexity compared to the backpropagation-based global learning hardware.

To realize energy-efficient hardware to solve the EP’s dynamic equation, Kendall et al. showed simple analog circuits with memristor synapses could solve the EP’s dynamic equation conceptually [18]. The memristors mean resistive-switching memories, where conductance can be programmed by applying voltage or current. There are many different kinds of materials and devices that can demonstrate resistive switching behaviors, for example, using [23,24,25]. For the fabrication and device technologies of memristors, a wide range of research, for example, peptide-based memristors and quantum-dot-based memristors, is still going on [23,24,25].

To develop memristor-based EP circuits with the weight-update controller, Seokjin et al. proposed a hardware-friendly weight-updating algorithm modified for the hardware implementation of EP’s algorithm [26]. In addition, Seokjin et al. developed memristor-based neural networks and a weight-update control circuit for solving the EP’s dynamic equation [26].

**Figure 1:**
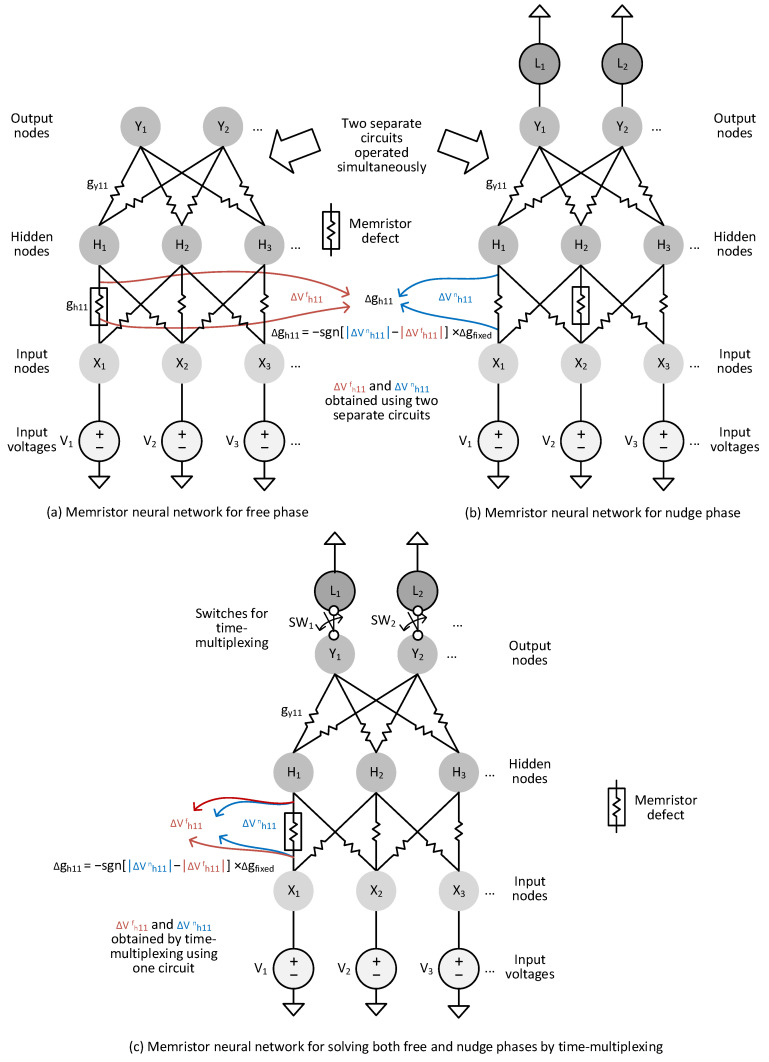
The conceptual diagrams of two memristor-based neural networks for solving the EP’s dynamic equation simultaneously in (**a**) the free phase and (**b**) the nudge phase using two separate circuits. (**c**) The diagram of one memristor-based neural network for solving both the free and nudge phases by time-multiplexing, where one memristor EP circuit is used for both the free and nudge phases instead of two separate circuits.

To explain in detail the previous memristor circuits, conceptual diagrams of memristor-based neural networks are shown in Figure 1a,b, which are for solving the EP’s equation in the free and nudge phases, respectively [12]. Figure 1a shows a conceptual memristor circuit for solving the EP’s algorithm in the free phase, where the circuit is composed of input voltages, input nodes, hidden nodes, and output nodes. The synaptic weights are implemented using memristors, as shown in Figure 1a. The input voltages are represented with V1, V2, etc. Similarly, the input, hidden, and output nodes are represented with X, H, and Y, respectively. gh11 is a synaptic weight between the input node, X1, and the hidden node, H1. Similarly, gy11 is a synaptic weight between the hidden node, H1, and the output node, Y1. As mentioned, gh11 and gy11 can be implemented by memristors. As shown in Figure 1a, the input voltages are connected to the input nodes. The input, hidden, and output nodes are connected layer by layer through synapses. In the free phase’s operation, the output nodes are left floating to be free from the labels. When the neural network’s state variables reach equilibrium in the free phase for a certain input vector, the network’s outputs can be regarded as the inference results for the corresponding input.

Figure 1b shows a conceptual memristor circuit for solving the EP’s dynamic equation in the nudge phase. One difference between the free and nudge phases is the output nodes are left floating or driven by the labels. In the free phase, the output nodes are left floating and not connected with the labels, as shown in Figure 1a. On the other hand, in the nudge phase in Figure 1b, when the network reaches equilibrium, the output nodes are applied by the labels. This leads to a difference in synapse voltages between the free and nudge phases that can be used for calculating the synaptic weight update, as stated as follows.

The weight update is calculated according to the difference in synapse voltages between the free and nudge phases [12]. For example, assume the synaptic weight update of gh11 between H1 and X1 in Figure 1a,b. Here, the free-phase and nudge-phase circuits should have the same weights. It means that gh11 in Figure 1a is the same as gh11 in Figure 1b. The synaptic weight update of gh11, Δgh11 can be calculated with the following Equation (2) [26]:

(2) $$∆g_{h11}=-sgn\left[\left|∆V_{h11}^{n}\right|-\left|∆V_{h11}^{f}\right|\right]\times {∆g}_{fixed}$$

Here, ‘sgn’ means a sign function of $\left|∆V_{h11}^{n}\right|-\left|∆V_{h11}^{f}\right|$ that can be either plus or minus. ${∆g}_{fixed}$ is a fixed change in the programming memristor’s conductance. $∆V_{h11}^{n}$ is a voltage drop of the memristor synapse, gh11, in the nudge-phase circuit in Figure 1b. Similarly, $∆V_{h11}^{f}$ represents a voltage drop of the memristor synapse, gh11, in the free-phase circuit in Figure 1a. As mentioned earlier, gh11 of the free-phase circuit should be the same as that of the nudge-phase circuit. However, $∆V_{h11}^{f}$ can be different from $∆V_{h11}^{n}$. This is because the output nodes of the nudge-phase circuit are applied by the labels such as L1 and L2, as shown in Figure 1b. On the contrary, the output nodes in Figure 1a are left floating and are not driven by the labels L1 and L2.

The memristor circuits for solving the EP’s dynamic equations in both free and nudge phases could successfully update the memristor synapses according to the modified EP algorithm [26]. The previous results indicated the neural network’s performance implemented by the memristor EP circuits was comparable to the neural networks based on the original EP algorithm [26].

However, still, memristor fabrication techniques are not mature even today; therefore, they may have some issues of defects and variability [27,28]. Considering the memristor’s defects and variability, the separation of free-phase and nudge-phase circuits can cause a serious degradation of the neural network’s performance. If the memristor circuit for solving the EP’s dynamic equation in the free phase is different from that of the nudge phase, the two separate circuits may suffer different defects and variability. If so, as they are trained by local learning, the two separate synapses implemented by two different memristors may suffer the problems of defects and variability more severely.

To overcome the defect and variability problems, this paper proposes a new time-multiplexing technique using one memristor circuit that can solve the EP’s dynamic equation of both the free and nudge phases sequentially, one by one, as conceptually shown in Figure 1c. In this figure, SW1 and SW2 are added to perform time-multiplexing. For solving the EP’s dynamic equation in the free phase, the switches are off. On the contrary, for the nudge phase, they are on. By using the one memristor circuit to solve both the free and nudge phases sequentially one by one, the variability and defect problems can be compensated for during the training time.

The reason why we should solve the free and nudge phases using the one memristor circuit can be explained as follows. Let us look at memristor defects represented with box symbols, as shown in Figure 1a,b. As mentioned earlier, the free and nudge circuits are shown in Figure 1a,b, respectively. If a memristor defect occurs at the synapse between X1 and H1 in the free phase circuit and the other defect is found between X2 and H2 in the nudge circuit, the two defects placed in different locations can affect the solutions of free and nudge phases differently. As the training epochs are increased, the weight update cannot reduce the error between the free and nudge solutions because the defect locations are different in the two circuits of Figure 1a,b. However, if the free and nudge solutions are obtained by the one memristor circuit in Figure 1c, the error between the free and nudge solutions can be reduced as the training epochs are increased. Let us look at the memristor defect between X1 and H1 in Figure 1c, which occurs at the same location for both free and nudge solutions. By doing so, the defect and variation problems in memristor crossbars can be compensated for automatically in the one memristor circuit in Figure 1c.

In addition, by uniting the free and nudge phases into one memristor circuit, we can reduce the area overhead of not only the memristor circuit for solving EP’s algorithm but also the weight-update control circuit. In the next section, the proposed memristor EP scheme that can solve both free and nudge phases using the time-multiplexing technique will be explained in detail.

## 2. Method

**Figure 2:**
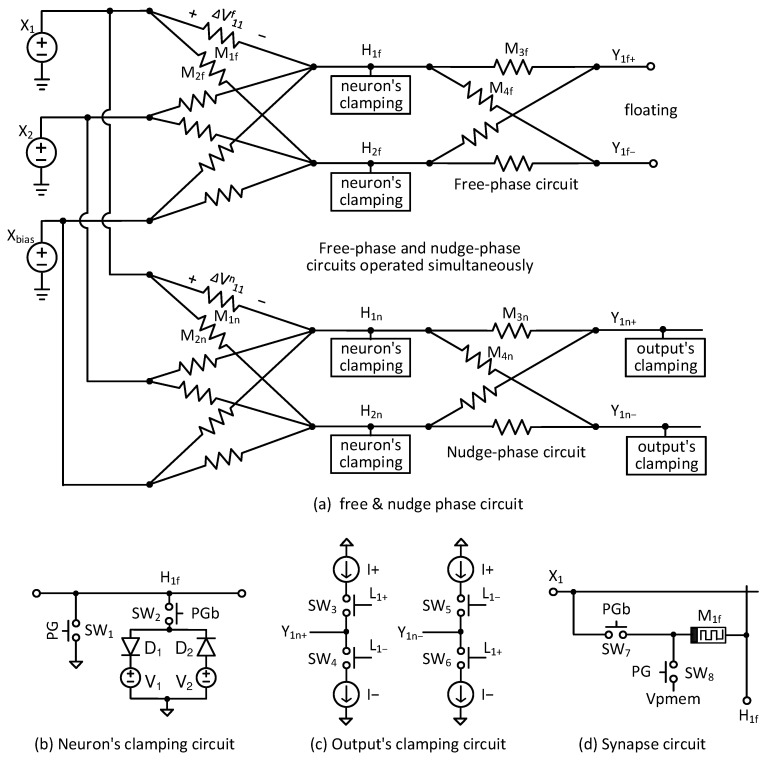
(**a**) The free-phase and nudge-phase circuits for solving the EP’s dynamic equation in the previous work; (**b**) the neuron’s clamping circuit; (**c**) the output’s clamping circuit; (**d**) the synapse circuit.

In this section, the previous technique is reviewed first [26]. In the technique, two circuits are used for solving the EP dynamic equation of free phase and nudge phase separately, as shown in Figure 2a [12,26]. Here, X1 and X2 are input voltages, and Xbias is a neural network’s bias voltage. One thing to note here is that X1, X2, and Xbias are applied to the two memristor circuits of free phase and nudge phase simultaneously. And the two memristor circuits of free phase and nudge phase should be identical [26]. Particularly, the upper in Figure 2a represents the free-phase memristor circuit for obtaining the free-phase solution of EP’s dynamic equation. Simultaneously, the lower in Figure 2a can calculate the nudge-phase solution of EP’s dynamic equation using the same input and bias voltages with the free-phase circuit.

Explaining Figure 2a in detail, H1f, H2f, etc., represent hidden nodes in the free-phase circuit. H1n, H2n, etc., represent hidden nodes in the nudge-phase circuit. Y1f+ and Y1f− are positive and negative voltages of output neurons, respectively, in the free-phase circuit. Y1n+ and Y1n− are positive and negative voltages of output neurons in the nudge phase. M1f is a synaptic weight between X1 and H1f neurons. Here, ΔVf11 is a voltage drop of memristor synapse M1f. M2f is a synaptic weight between X1 and H2f neurons. Similarly, M3f and M4f are synaptic weights between H1f and Y1f+ and between H1f and Y1f−, respectively. M1n and M2n are synaptic weights between the input and hidden neurons in the nudge-phase circuit. ΔVn11 is a voltage drop of memristor synapse M1n. M3n and M4n represent synapses between the hidden and output neurons in the nudge phase.

The hidden-node voltages of H1f, H1n, etc., should act like a ReLU activation function. To do so, a neuron’s clamping circuit shown in Figure 2b is used for the hidden nodes of both free-phase and nudge-phase circuits [18]. The neuron’s clamping circuit uses an anti-parallel diode configuration for the implementation of the nonlinear ReLU function. In Figure 2b, D1 is connected to V1, and D2 is connected to V2. When the hidden voltage becomes very high, D1 is turned on. By doing so, the hidden voltage can be limited by V1. On the other hand, when the hidden node becomes too low, D2 is turned on, and the node is limited by V2. This voltage transfer behavior can be considered very similar to the ReLU function. Figure 2b has SW1 and SW2. SW1 and SW2 are turned on and off, respectively, when PG is high. At that time, the hidden node is connected to the ground, and the neuron’s clamping circuit is disabled. When PG is low, SW1 and SW2 are turned off and on to make the clamping circuit ready to work. Here, PG becomes high only during the programming time of memristor synapses.

An output’s clamping circuit is indicated in Figure 2c. As mentioned, the output nodes in the free-phase circuit in Figure 2a should be left floating to be free from the output’s labels. On the contrary, the output nodes in the nudge phase in Figure 2a should be driven by the output’s labels. To do this, the output’s clamping circuit in Figure 2c is composed of current sources and switches, which are controlled by labels such as L1+ and L1−. Y1n+ and Y1n− are positive and negative output voltages from the nudge-phase circuit in Figure 2a. In Figure 2c, I+ and I− mean positive and negative current sources, respectively. The magnitudes of I+ and I− are identical, but their signs are opposite. When L1+ is high and L1− is low, SW3 and SW6 are turned on, while SW4 and SW5 are turned off. Conversely, when L1+ is low and L1− is high, Y1n+ and Y1n− are the opposite of the previous case. By controlling SW3−SW6, the output nodes in the nudge-phase circuit are driven by positive or negative sources according to the labels.

Figure 2d shows a synaptic circuit including switches for memristor’s programming. Here, SW7 and SW8 are the switches for programming the memristor M1f. The M1f means the memristor synapse between X1 and H1f. When PG is high and PGb is low, the memristor M1f can be programmed by the programming pulse of Vpmem. On the other hand, when PG is low and PGb is high, the memristor M1f works as a synaptic weight.

**Figure 3:**
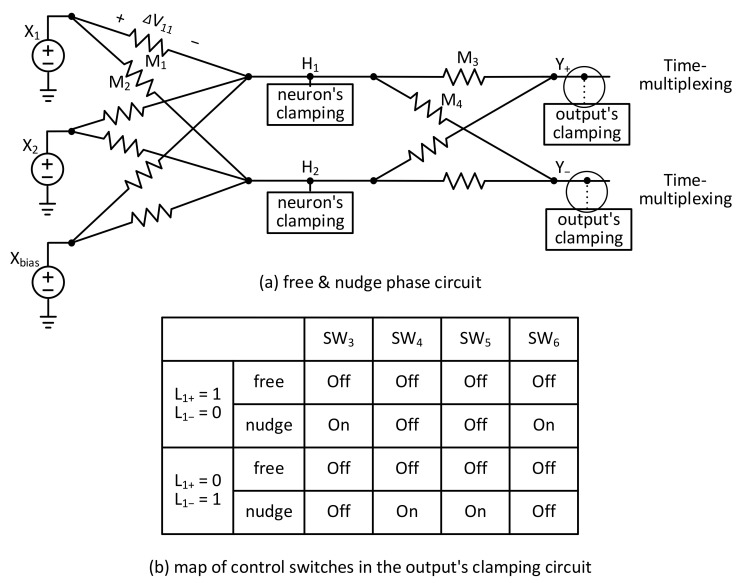
(**a**) The schematic of the time-multiplexed memristor circuit for calculating EP’s solution of the free and nudge phases sequentially. (**b**) The map of the control switches in the output’s clamping circuit for solving the EP’s dynamic equation in the free and nudge phases sequentially.

As shown in Figure 2a, if the EP algorithm is solved separately using the two memristor circuits, the free-phase and nudge-phase solutions can be affected by different defects and variability due to the two different memristor crossbars of free and nudge phases. To avoid this problem, a time-multiplexing technique can be considered to solve the EP dynamic equation of free phase and nudge phase using only memristor crossbar one by one. To do so, Figure 3a shows a time-multiplexed memristor circuit for solving EP’s dynamic equation in the free and nudge phases sequentially. Here, X1 and X2 are input voltages. Xbias means a bias voltage. H1 and H2 represent hidden node voltages. Unlike Figure 2a, the hidden nodes are common to both the free and nudge phases in Figure 3a. Y1+ and Y1− are positive and negative output nodes, respectively. M1 and M2 are synaptic weights between the input and hidden neurons in Figure 3a. M3 and M4 represent synapses between the hidden and output neurons. Here, when the free-phase solution is calculated, the output clamping circuit is disconnected from the output nodes. On the contrary, the output’s labels are connected to the output nodes for solving the nudge phase’s equation. The switching between the free and nudge phases is performed by controlling the switches in the output’s clamping circuit, as explained in the following.

In Figure 3b, for time-multiplexing of the free and nudge phases, a map of control switches in the output’s clamping circuit is shown. The control switches in the output’s clamping circuit are shown in Figure 2c. For given labels, the memristor circuit in Figure 3a solves the EP dynamic equation for the free and nudge phases sequentially using the time-multiplexing operation. First, during the free phase, the output nodes of Y1+ and Y1− are left floating regardless of the labels of L1+ and L1−. To do so, all the control switches of SW3-SW6 are turned off. After calculating the solution of the free phase, the memristor circuit is re-configured to obtain the nudge-phase solution. The reconfiguration is performed by changing the control switches in the output’s clamping circuit. When L1+ = 1 and L1− = 0, SW3 and SW6 are turned on and SW4 and SW5 are off. On the other hand, when L1+ = 0 and L1− = 1, SW4 and SW5 are on, and SW3 and SW6 are off.

**Figure 4:**
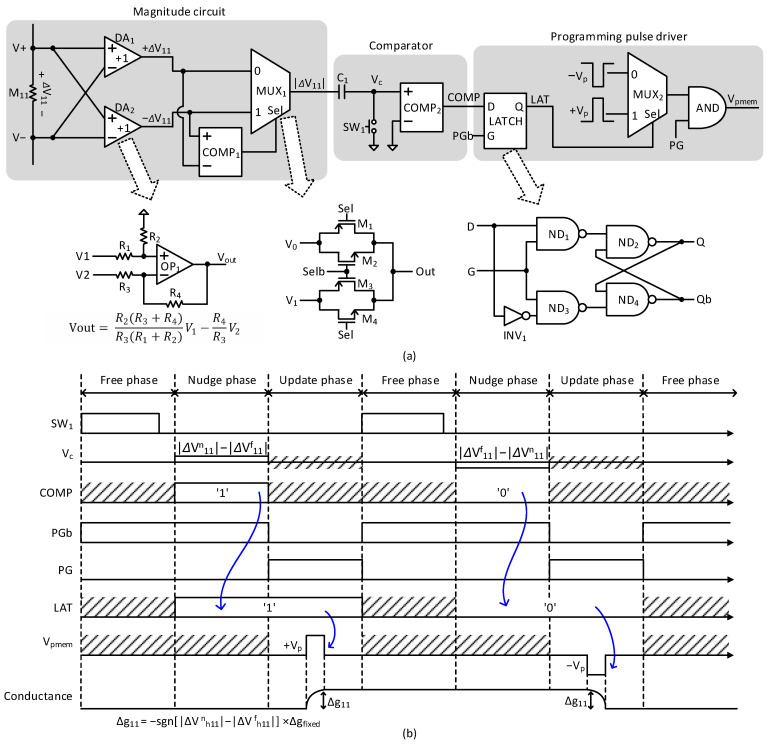
(**a**) The schematic of the weight-update control circuit of memristor synapses. (**b**) The operational timing diagram of the weight-update control circuit of memristor synapses.

Figure 4a shows a schematic of the weight-update control circuit of memristor synapses. The weight-update control circuit is composed of three parts. They are the magnitude circuit, comparator, and programming pulse driver, respectively, as shown in Figure 4a. First, in the magnitude circuit, a voltage drop of memristor synapse, ΔV11, is applied to two different amps, DA1 and DA2. The difference amp is shown also in Figure 4a. In the difference amp, OP1 means operational amplifier. Similarly, R1, R2, R3, and R4 are resistors used in the difference amplifier. If R1 = R3 and R2 = R4, the difference amp delivers $\frac{R_{2}}{R_{1}}\left(V_{1}-V_{2}\right)$ for its output. Here, the DA1 and DA2 are applied by plus ΔV11 and minus ΔV11, respectively. As a result, the DA1 and DA2 can generate $+\frac{R_{2}}{R_{1}}{∆V}_{11}$ and $-\frac{R_{2}}{R_{1}}{∆V}_{11}$, respectively, for the outputs. Here, if R1 = R2, the outputs of DA1 and DA2 are +ΔV11 and −ΔV11.

COMP1 compares +ΔV11 and −ΔV11. The COMP1 can be designed using a simple OP amp circuit. For the COMP1, if the plus input is larger than the minus one, +ΔV11 is transferred to the next part by MUX1. In the opposite case, −ΔV11 is transferred to the second part. By doing so, only the magnitude of ΔV11 can selected by MUX1, regardless of the polarity. The MUX1 can be designed using simple transmission gate circuits, as shown in Figure 4a. If ‘Sel’ = 0, V0 is transferred to the output of MUX1. If ‘Sel’ = 1, V1 is transferred to the output of MUX1.

The second part of Figure 3a is composed of C1, SW1, and COMP2. Here, C1 stores the magnitude of ΔV11 in the free phase. SW1 controls the time-multiplexing of free and nudge phases. During the free phase, the magnitude of ΔV11 can be stored at C1 by turning on SW1. Here, the magnitude of ΔV11 in the free phase is denoted by |ΔVf11|. At the following nudge phase, MUX1’s output becomes as high as |ΔVn11|. Here, |ΔVn11| means the magnitude of ΔV11 in the nudge phase. When |ΔVn11| > |ΔVf11|, COMP2’s output becomes high. On the other hand, when |ΔVn11| < |ΔVf11|, COMP2’s output is low. COMP2 means a comparator circuit and can be designed by a simple OP amp circuit like COMP1.

The third part of Figure 3a is composed of LATCH, MUX2, and gates. The latch is controlled by ‘PGb’. When ‘PGb’ is high and low, the latch is transparent and opaque, respectively. COMP2’s output is latched in the third part of Figure 3a according to the comparison result of the free and nudge phases. The latched output signal of LAT selects a positive or negative programming pulse by MUX2. The positive and negative programming pulses are represented by +Vp and –Vp in Figure 3a, respectively. MUX2’s output and ‘PG’ enter the AND gate. By doing so, the programming pulse can be applied to memristor synapses only when ‘PG’ is high. The AND gate’s output is ‘Vpmem’. The LATCH circuit is also shown in Figure 4a, where a simple SET-RESET latch is used. In the LATCH circuit, ND means a NAND gate, and INV means an INVERTER gate.

Figure 4b shows an operational timing diagram of the weight-update control circuit of memristor synapses. The operation of the weight-update control circuit can be explained using three phases. They are free, nudge, and update phases, respectively, as shown in Figure 4b.

In the free phase, the magnitude circuit delivers |ΔVf11| to C1. At this time, it should be noted that ‘Vc’ is zero because SW1 is turned on. In the following nudge phase, the magnitude circuit delivers |ΔVn11| to C1. In this case, ‘Vc’ becomes as high as |ΔVn11| − |ΔVf11| because SW1 is turned off in the nudge phase. COMP2 compares ‘Vc’ with respect to 0V. Thus, if ‘Vc’ > 0, COMP2’s output becomes high. If ‘Vc’ < 0, COMP2’s output becomes low. The comparator’s output is latched when ‘PGb’ is low. The latched output signal of LAT selects a positive or negative programming pulse by MUX2. The positive and negative programming pulses are represented by ‘ + Vp’ and ‘–Vp’ in Figure 4a, respectively. The MUX2’s output and ‘PG’ enter the AND gate. By doing so, the programming pulse can be applied to memristor synapses only when ‘PG’ is high. If ‘Vpmem’ is positive, the memristor’s conductance is increased. On the contrary, if ‘Vpmem’ is negative, the memristor is programmed to decrease its conductance.

In the timing diagram of Figure 4b, the free and nudge phases are as long as 10 us, respectively. The weight-update phase is 10 us. Actually, no timing errors were found in the circuit simulation because the free, nudge, and update phases are long enough to avoid the timing problem. One more thing to note is that the LATCH sampling ‘COMP’ signal is driven by ‘PGb’, and the AND gate delivering ‘Vpmem’ is driven by ‘PG’, as shown in the programming pulse driver in Figure 4a. This is a typical master–slave architecture like a simple D-flipflop circuit that is well-known for its stable timing operation. When ‘PGb’ and ‘PG’ become low and high, respectively, ‘LAT’ signal, which is the output of LATCH, controls the MUX2. At the same moment, the AND gate is enabled by ‘PG’ and can deliver ‘Vpmem’ according to ‘+Vp’ or ‘-Vp’ on its input terminals. Here, ‘+Vp’ and ‘-Vp’ are the positive and negative programming pulses for changing memristor’s conductance. ‘+Vp’ and ‘-Vp’ can be delivered to memristors only after ‘PG’ becomes high. By doing so, the timing problem of programming voltage pulses can be avoided thoroughly, as shown in Figure 4b.

One more thing to explain is the precision of conductance change per programming pulse. In the weight update control circuit, the voltage amplitude of programming pulse is fixed by ‘Vp’. Here, the voltage amplitude of ‘Vp’ is defined by the minimum voltage amplitude that can change memristor’s conductance by $∆g_{fixed}$. Using the fixed ‘Vp’, the update control circuit only decides the positive or negative pulse of ‘Vp’ which can increase or decrease memristor’s conductance, respectively, as shown in Figure 4b. The conductance change equation was already explained in Equation (2) in the introduction part. If the voltage amplitude of ‘Vp’ is low enough to make $∆g_{fixed}$ very small, the memristor’s conductance can be programmed precisely, for example, as accurately as 6–8 bit resolution [29].

## 3. Results

**Figure 5:**
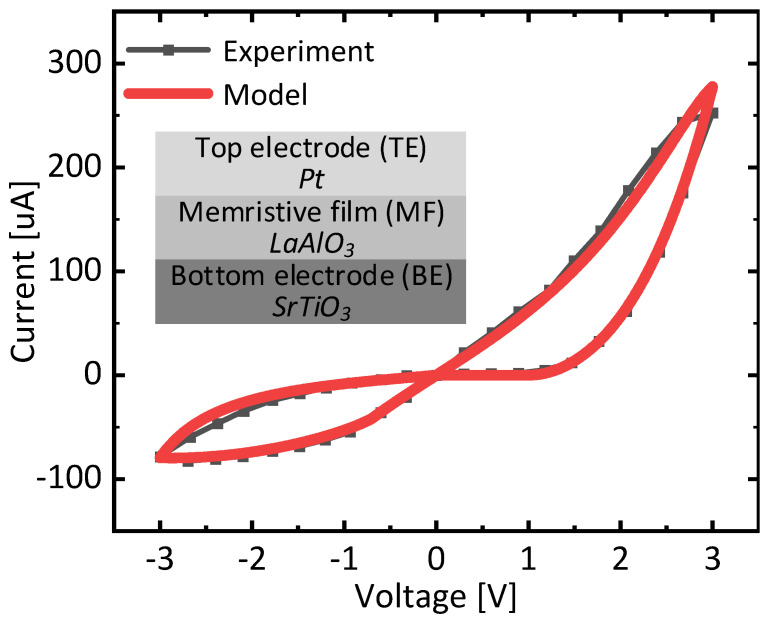
The butterfly curves of measurement and modeling of memristors with the device’s cross-sectional view.

Figure 5 shows butterfly curves of measurement and verilog-a modeling of memristors with a memristor’s cross-sectional view observed in this paper. The device has a top electrode, a memristor film, and a bottom electrode. They are made from Pt, LaAlO3, and StTiO3, respectively [30]. Here, the experimental and modeling data are shown in black and red lines, respectively. The modeling is performed using verilog-a language, and the simulation tool used in this paper is CADENCE SPECTRE. The modeling equations used in this simulation are explained in detail in the following reference [31,32,33]. The measurement data in Figure 5 were obtained by Keithley 4200 semiconductor parameter analyzer and probing station. The weight-update control circuit in Figure 4a is also simulated using the CADENCE SPECTRE with CADENCE GPDK 45-nm CMOS parameters to verify its timing operation. From Figure 5, it is observed that the verilog-a model used in this paper matches the measurement well [30]. The accurate verilog-a modeling of memristors is very important for performing the accurate simulation of memristor circuits [31,32,33].

The proposed memristor circuits in this paper should be verified for various neural networks’ datasets. To do so, we chose only the MNIST and CIFAR10 datasets to test the proposed memristor circuits in this paper. Of course, the more complicated dataset, such as IMAGENET, can also be considered here. However, the circuit simulation time for testing the proposed memristor circuits using the IAMGENET dataset takes too long to finish the simulation within a certain reasonable time. Thus, only MNIST and CIFAR10 datasets are used to train the proposed memristor circuits in this paper.

First, using the MNIST dataset, the proposed memristor EP scheme is compared with the previous scheme, which separates the free phase circuit from the nudge phase one, as shown in Figure 2a. Here, it is assumed that the memristor under the simulation has defects and variability. The MNIST dataset consists of 60,000 training vectors and 10,000 testing ones. For testing the MNIST dataset, the memristor EP circuits are designed with 400 input and 256 hidden neurons and numerous memristor synapses connecting them. The circuit simulation was performed using the Python program run with the Ngspice circuit simulator [34,35,36]. Here, the memristor circuit’s netlist was extracted from CADENCE SPECTRE, and the extracted netlist was simulated for the MNIST training and test dataset using the Python program run with the Ngspice simulator [34,35]. One thing to note here is that we used the training dataset of only 3 classes among the total 10 classes for the circuit simulation. This is to reduce the circuit simulation time because the circuit simulation time is proportional to the training dataset size. If we train the memristor circuits for the training dataset of three classes, the circuit simulation time can be reduced by as much as 70% because the total training dataset is composed of 10 classes.

**Figure 6:**
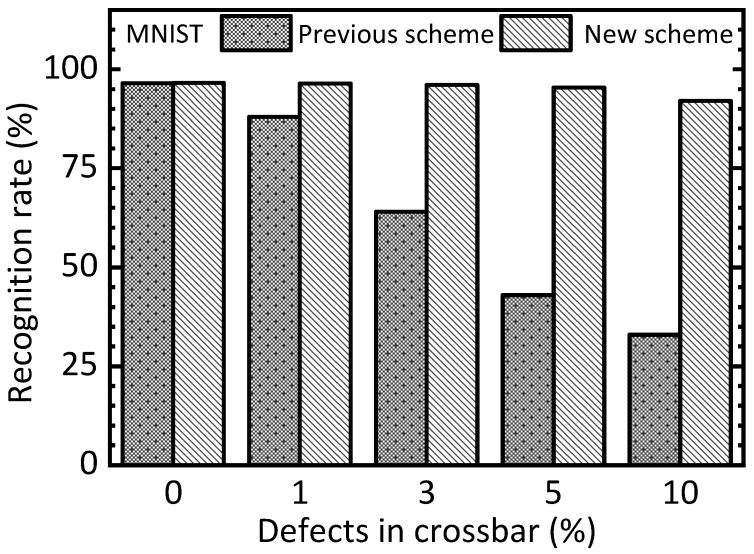
The simulated MNIST recognition rate of the previous and new schemes with increasing the percentage of defects in the memristor crossbar.

Figure 6 compares the previous two-circuit scheme and the new time-multiplexed scheme in terms of the recognition rate of the MNIST test dataset. In Figure 6, the *y*-axis represents the MNIST recognition rate that is obtained from the Ngspice circuit simulation. The *x*-axis means the percentage of defects in the memristor crossbar. If there is no defect in the memristor crossbar, the previous and new schemes indicate almost the same rates, which are 96.5% and 96.6%, respectively. However, as the defect percentage increases, the gap in recognition rate between the previous and new schemes becomes more significant. In the case of the previous scheme, the recognition rate significantly decreased from 96.5% to 33%, when the defect percentage increased from 0% to 10%. Unlike the previous scheme, the recognition rate of the new scheme is degraded little in terms of the spice of the defect percentage, which increased from 0% to 10%. For the defect percentage as high as 10%, the rate is still 92%, which is degraded only by 4.6% compared to the defect percentage of 0%.

This result demonstrates the new scheme is more resilient to memristor defects. The proposed scheme can effectively compensate for the neural network’s performance degradation due to the memristor defects. This is because the free and nudge phases are solved by the same memristor circuit in the new scheme. On the contrary, in the previous scheme, the defects in the free-phase circuit can happen in different memristors from the nudge-phase circuit, resulting in significant errors when calculating the weight updates according to Equation (2). As the epochs are increased, the errors in the previous scheme are accumulated, thereby degrading the neural network’s performance significantly. However, in the new scheme, the free and nudge phases are solved by the same memristor circuit. In this case, the errors caused by the memristor defects can be recovered by the training.

**Figure 7:**
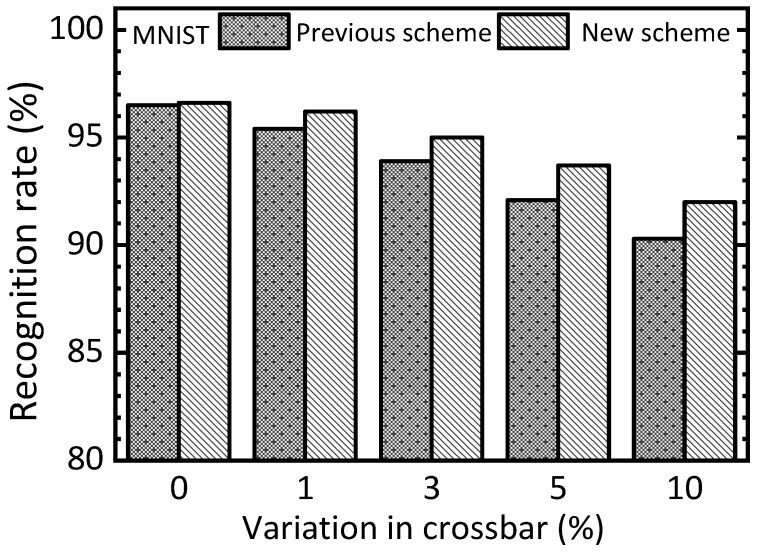
The simulated MNIST recognition rate of the previous and new schemes with increasing the percentage of variation in the memristor crossbar.

Figure 7 compares the previous and new schemes with increasing the percentage of variation in the memristor crossbar. When the percentage of variation is zero, the previous and new schemes indicate the recognition rates of 96.5% and 96.6%, respectively. The gap between the two schemes is as little as 0.1%. When the percentage of variation becomes as large as 10%, the previous and new schemes demonstrate rates of 90.3% and 92.1%, respectively. For the 10% variation, the gap between the two schemes is observed as large as 1.8%.

One more thing to note here is that the new scheme shows recognition rates of 92% and 92.1%, respectively, for the defect percentage = 10% and the variation percentage = 10%. The little gap between the defect percentage = 10% and variation percentage = 10% means both the defect and variability problems can be recovered well in the new scheme. However, the previous scheme indicates rates of 33% and 90.3% for the defect percentage = 10% and the variation percentage = 10%, respectively. The reason why the previous scheme shows a very big degradation of recognition rate when the defect percentage = 10% is that the defects placed in different cells between the free and nudge circuits are very critical to the neural network’s performance. On the contrary, the variation percentage = 10% can be better to be recovered even for the previous scheme.

In addition to the MNIST dataset, the proposed memristor circuits are also verified using the CIFAR10 dataset in this paper. Like Figure 6 and Figure 7, the memristor simulated is assumed defect and variability. The CIFAR10 dataset consists of 50,000 training vectors and 10,000 testing ones. For training the CIFAR10 dataset, if we implement the entire neural network using the memristor EP circuits, the circuit simulation time can be very long. To shorten the circuit simulation time, the convolution layers are modeled using Python, and only the fully connected layers are implemented by the memristor EP circuits. By doing so, the circuit simulation time can be reduced significantly. Specifically, for testing the CIFAR10 dataset, the EP’s memristor circuits designed in this paper have 512 input and 256 hidden neurons. Similar to the MNIST training, the training dataset of only 3 classes among the total 10 classes is used in the circuit simulation. This is to reduce the circuit simulation time because the circuit simulation time is proportional to the training dataset size. If we train the memristor circuits for the training dataset of only three classes, the circuit simulation time can be reduced by as much as 70%.

**Figure 8:**
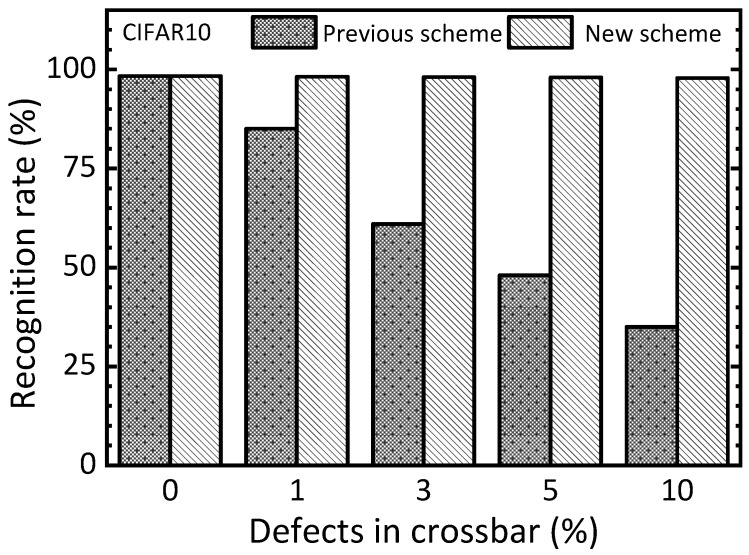
The simulated CIFAR10 recognition rate of the previous scheme and the new one in terms of increasing the defect percentage in the memristor crossbar.

Figure 8 compares the previous two-circuit scheme and the new time-multiplexed scheme in terms of the recognition rate of the CIFAR10 test dataset with varying defect percentages. When the defect percentage is zero, both the previous scheme and the new one indicate a rate of 98.3%. As the defect percentage increases to as high as 10%, the previous scheme and the new one show 35% and 97.8%, respectively. This large gap between the previous scheme and the new one clearly indicates that the new scheme can improve the defect tolerance significantly more than the previous one.

**Figure 9:**
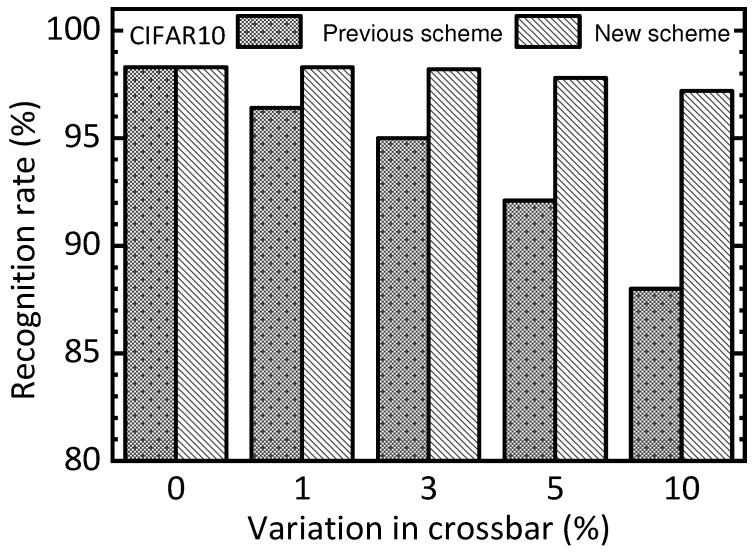
The simulated CIFAR10 recognition rate of the previous scheme and the new one in terms of increasing the percentage of variation in the memristor crossbar.

Figure 9 compares the previous scheme and the new one for the CIFAR10 test dataset in terms of increasing the variation percentage in the crossbar. When the variation percentage is zero, both the previous and new schemes show a recognition rate of 98.3%. When the variation becomes as high as 10%, the previous scheme and the new one are 88% and 97.2%, respectively. Similar to Figure 8, Figure 9 demonstrates obviously that the new scheme can be more robust in terms of device variation than the previous one.

Let us explain the simulation in more detail with various percentage numbers of defects and variations, as shown in Figure 6, Figure 7, Figure 8 and Figure 9. In this simulation, we used a random number generation function provided in the Python mathematical library. By doing so, many synaptic weight sets of EP memristor circuits are obtained to consider the various cases of defects and variations, such as 3%, 5%, and 10%, as indicated in Figure 6, Figure 7, Figure 8 and Figure 9. Explaining the statistical analysis, for example, the defect percentage = 10% can be assumed. To calculate the recognition rate for the 10%, in this paper, five weight sets with different random defects are obtained using five different random seed numbers. Here, the defect maps are different for the five weight sets, though they have the same defect percentage = 10%. The final recognition rate is calculated by averaging the five recognition rates that are obtained from the five defect maps, respectively. One thing to note here is that the gap between the highest rate and the lowest one is less than 0.3% for the defect percentage = 10%. Similarly, for the other cases, the gaps between the highest and lowest are so small that they can be ignored, too.

One more thing to note here is that the proposed memristor circuits were verified using different HRS and LRS values. Here, HRS and LRS mean high resistance state and low resistance state, respectively. This is to verify that the proposed memristor EP circuits can work well for a wide range of LRS and HRS values. For Figure 6, Figure 7, Figure 8 and Figure 9, HRS and LRS are 1 mega-ohm and 10 kilo-ohm, respectively. In addition, HRS = 500 kilo-ohm and LRS = 10 kilo-ohm are tested. And HRS = 300 kilo-ohm and LRS = 10 kilo-ohm are also verified by the circuit simulation. For these three cases of different HRS and LRS values, the recognition rates simulated are almost the same, exhibiting a difference in the simulated rates of less than 2%.

For the defect and variation problems, we verified the proposed memristor EP circuits using the circuit simulation with the MNIST and CIFAR10 datasets in Figure 6, Figure 7, Figure 8 and Figure 9. In addition to the memristor defect and device variation issues, the cycle-to-cycle variation can be considered, too. However, fortunately, the cycle-to-cycle variation can be neglected in this paper. As shown in Figure 4b, the free and nudge phases are consecutive. This means the nudge phase starts just after the free phase. The time gap between the free and nudge phases is shorter than 10 μs in this work. This short timing gap between the two phases may cause very little cycle-to-cycle variation that can be negligible [29].

Finally, the overhead area of the new scheme should be discussed and compared to that of the previous scheme. By uniting the free and nudge phases into one memristor circuit, the new scheme can reduce the area overhead of not only the memristor circuit for solving EP’s algorithm but also the weight-update control circuit. For the area of the memristor synapse circuit, the new scheme needs only one memristor synapse circuit that can solve both the free and nudge phases by time-multiplexing. On the contrary, the EP’s dynamic equation is solved by the two circuits for the free and nudge phases, respectively, in the previous scheme. Therefore, the area of the memristor synapse circuits is almost double that of the previous scheme. For the area of the weight-update control circuit, the previous scheme has four op amps, three comparators, and three MUXs. On the other hand, the new scheme needs only two op amps, two comparators, two MUXs, and one capacitor. As a result, roughly, the area of the weight-update control circuit in the new scheme is reduced by 1/3 of that in the previous scheme.

## 4. Conclusions

Local learning algorithms, such as Equilibrium Propagation (EP), have emerged as alternatives to global learning methods like backpropagation for training neural networks. The EP algorithm offers the potential for more energy-efficient hardware implementation by utilizing only local neuron information for weight updates. However, the immature fabrication processes of memristors, which result in defects and variability issues, have been significant challenges in the practical realization of EP hardware.

We introduced a novel time-multiplexing technique that combined the free and nudge phases of EP into a single memristor circuit, overcoming the limitations of previous implementations that used two separate circuits for each phase. By solving the dynamic equation of both phases using the single circuit, our method can compensate for memristor defects and variability during the training process.

The effectiveness of our approach was demonstrated through simulations using the MNIST dataset. Even with a 10% defect percentage in memristors, the new scheme maintained a 92% recognition rate, a significant improvement over the previous scheme, which achieved only 33% under the same condition. Moreover, the proposed scheme could significantly reduce the area overhead of both the memristor circuit for solving EP’s algorithm and the weight-update control circuit.

## Data Availability

The data presented in this study are available on request from the corresponding author.
